# Cardiovascular Risk Factors in Hemodialysis Patients: Results from Baseline Data of Kaleidoscopic Approaches to Patients with End-stage Renal Disease Study

**DOI:** 10.2188/jea.15.96

**Published:** 2005-06-01

**Authors:** Masaki Ohsawa, Karen Kato, Kazuyoshi Itai, Toshiyuki Onoda, Ryuichiro Konda, Tomoaki Fujioka, Motoyuki Nakamura, Akira Okayama, the KAREN Study Group

**Affiliations:** 1 Department of Hygiene and Preventive Medicine, School of Medicine, Iwate Medical University.; 2 Department of Urology, School of Medicine, Iwate Medical University.; 3 Second Department of Internal Medicine, School of Medicine, Iwate Medical University.; 4 Department of Preventive Cardiology, National Cardiovascular Center.

**Keywords:** the KAREN Study, Renal Dialysis, Risk Factors, Population, Cross-Sectional Studies

## Abstract

BACKGROUND: The prevalence of cardiovascular risk factors and the prevalence of comorbidities in adult hemodialysis patients in Japan are not fully understood.

METHODS: In “Kaleidoscopic Approaches to Patients with End-stage Renal Disease Study” (The KAREN Study, 2003), trained research stuff examined 1,214 adult hemodialysis patients (mean age, 61.2 years; 779 males and 435 females) of 1,506 patients in northern areas of Iwate Prefecture. Cardiovascular risk factors and the prevalence of comorbidities in hemodialysis patients were compared with those in the general population using direct age-adjustment methodology and standardized morbidity ratios (SMRs).

RESULTS: In hemodialysis patients, common causes of end-stage renal disease were chronic glomerulonephritis (29.8%), diabetic nephropathy (24.5%), and other diseases. Prevalence and SMR of myocardial infarction were 5% and 9.6, respectively, and those of stroke were 13% and 5.7. The prevalences of hypertension and diabetes mellitus were 87% and 29%, respectively. Mean systolic blood pressure and mean diastolic blood pressure were 155 mmHg and 85 mmHg, respectively. Mean levels of total serum cholesterol, high-density lipoprotein cholesterol, and albumin in patients with end-stage renal disease were lower than those of the general population (160.6 vs. 203.3 mg/dL, 48.5 vs. 59.7 mg/dL, and 3.7 vs. 4.4 g/dL, respectively). Mean levels of C-reactive protein were higher than those of the general population (3.80 vs. 1.16 mg/L).

CONCLUSION: Hemodialysis patients have a high prevalence of cardiovascular risk factors and comorbidities. Levels of nutrition-related markers were lower, and C-reactive protein levels were higher, in hemodialysis patients than in the general population.

More than 200,000 patients (1,722 per million) with end-stage renal disease (ESRD) underwent maintenance renal replacement therapy in Japan in 2002.^1^ In the United States, the prevalence of patients with ESRD was 1,403 per million in 2001.^2^ The incidence and prevalence of ESRD, especially in diabetic and elderly patients, have been increasing over the past two decades in both countries.^1^^,^^2^

ESRD patients have a high mortality rate. The crude annual mortality rate of patients with ESRD has remained unchanged for the last ten years in Japan, at around 9%.^1^ The high mortality rate of patients with ESRD is partly attributable to their high incidence of cardiovascular disease (CVD).^3^

Little is known, however, about CVD risk factors in Japanese ESRD patients. One population-based study in ESRD patients was carried out more than ten years ago and reported that the prevalence of coronary artery disease in patients with ESRD was 1.4% in the 1970’s and 2.5% in the 1980’s.^4^^,^^5^ Prevalences of CVD risk factors and cardiovascular comorbidities in recent years, however, have not been determined despite an increase in the numbers of diabetic and elderly patients.

The aim of this study was to reveal the prevalence of CVD risk factors in hemodialysis patients using a population-based study. We also compared the prevalence of CVD risk factors in hemodialysis patients with those of the general population.

## METHODS

### Setting of the Study

We have conducted the “Kaleidoscopic Approaches to patients with end-stage RENal disease Study” (the KAREN Study). The KAREN Study is a population-based prospective study designed to determine the effects of risk factors on CVD morbidity and mortality in ESRD patients. The study region is a section of northern Iwate Prefecture located in the northern part of the main island of Japan. The study area consists of 38 municipalities with a total population of 939,448 in 2002. There are 26 dialysis institutes in this region.

A preliminary survey to determine the number of dialysis patients in this area was carried out by sending facsimiles or letters to 26 dialysis institutes in April 2003. All the 26 institutes informed us of their numbers of ESRD patients, which totaled 1,506 adult hemodialysis patients. The prevalence of hemodialysis was 1,596 per million, and 6% of ESRD patients were undergoing peritoneal dialysis. Directors of 25 institutes, in which 1,499 hemodialysis patients were undergoing hemodialysis therapy, agreed to participate in the study.

Initial investigations for the KAREN study began in June 2003 and finished in March 2004. Annual checks of patients’ medical records were scheduled to ascertain interim cardiovascular and cerebrovascular events, and will be continued for at least five years. This study was approved by the Medical Ethics Committee of Iwate Medical University and conducted in accordance with the guidelines of the Declaration of Helsinki.

### Subjects

We used baseline data from the KAREN Study for cross-sectional analysis. There were 1,499 adult hemodialysis patients in the KAREN Study, though we were not able to contact 52 of these patients because of serious physical conditions or mental disorders. We obtained written informed consent for participation in the study from 1,260 patients (acceptance rate: 87.1%). Baseline examinations were not conducted in 21 of the 1,260 patients because of deterioration in their general conditions. In the end, we enrolled 1,239 patients in our study.

In the cross-sectional analysis, we excluded data from 25 patients because blood samples were not obtained. Data from 1,214 patients (80.6% of the total patients, aged 22 to 95 years, 779 males and 435 females) were used for analysis.

### Research Staff and Data Collection

The KAREN staff includes two physicians (an urologist and a cardiologist), eight nurses, and 22 assistants. Assistants were recruited on an area-by-area basis, and they were involved in obtaining informed consents, checking questionnaires, and measuring blood pressure and body height. All the research staff were trained and approved before conducting the survey. A coordinating center was set up in the Department of Hygiene and Preventive Medicine, School of Medicine, Iwate Medical University, Morioka City, Iwate Prefecture, Japan.

Paper forms were brought to the coordinating center by the KAREN staff. Blood test data were sent to the coordinating center electronically, and only staff with permission was able to enter the room and edit data.

### Initial Examinations

The baseline examination consisted of a questionnaire, measurements of blood pressure and anthropometric data, medical information reviews, and blood tests.

#### (1) Questionnaire

Each participant was asked to complete a questionnaire during a hemodialysis session. The questionnaire consisted of 24 questions regarding past history, family history, medication history, alcohol drinking habits, smoking habits, sleeping time per day, occupational status, the number of housemates, food preferences, and self-assessment of personality. The KAREN staff helped disabled patients fill out questionnaires, without manipulation of responses.

#### (2) Blood Pressure and Anthropometric Data

KAREN research staff took all measurements of body height and blood pressure. Body weight was measured using an automated scale at each institute before dialysis. Body height was measured as the length from between the heels to the centriciput point, in the supine position, using a metallic tape measure. Blood pressure was measured in the contralateral arm in patients with patent arteriovenous fistulae or grafts. Pre-dialysis blood pressure was measured twice in the supine position using an automatic device (BP-103i II Model 513000, Nippon Colin, Komaki, Japan) after a five-minute bed rest prior to cannulation. Post-dialysis blood pressure was measured in the supine position in a similar manner after a five-minute bed rest immediately following removal of the cannulae. Systolic blood pressure (SBP) and diastolic blood pressure (DBP) were each calculated as the mean of two measurements. Body mass index (BMI) was calculated as dry weight (kg) divided by the square of body height (m).

#### (3) Reviews of Medical Records

The two physicians and eight nurses visited the 25 institutes and reviewed patients’ medical records and treatment regimens. They recorded patients’ characteristics such as age, sex, past history, family history, date hemodialysis was initiated, length of hemodialysis sessions, number of hemodialysis sessions per week, prescribed dry weight, inter-dialysis weight gain at the beginning of the week, cause of ESRD, diabetic status (based on past or current use of hypoglycemic agents), previous extremity amputation, comorbid conditions, current medications, falls in blood pressure (both falls in SBP of 30 mmHg or more and falls in SBP to below 90 mmHg) during hemodialysis sessions or the use of a vasopressor agent during hemodialysis sessions, blood pressure elevation (elevation in SBP of 30 mmHg or more) during hemodialysis sessions, use of erythropoietin, and other hemodialysis regimens.

#### (4) Blood Test Data

Pre-dialysis blood sampling was carried out at the beginning of hemodialysis sessions by the dialysis nursing staff. Blood samples were drawn from arteriovenous fistulae or grafts through dialysis cannulae into vacuum tubes containing EDTA or a serum separator gel or citrate. The blood samples were transported to a laboratory (Mitsubishi Kagaku Bio-Clinical Laboratories, Inc., Morioka branch office) and analyzed the same day.

Levels of total cholesterol, triglyceride, uric acid, and creatinine were measured by enzymatic assays. The urease GLC method was used to determine levels of Blood urea nitrogen (BUN). High-density lipoprotein (HDL) cholesterol levels were determined by a direct quantitative assay, while concentrations of sodium ion, chloride ion, and potassium ion were determined using electrodes. Serum levels of calcium were determined by the o-cresolphthalein complexone method. Total protein levels were determined by the biuret method, and serum albumin levels were determined by the bromcresol green method. All of the above biochemical data were analyzed using an automated analyzer (AU5232, Olympus Corp., Tokyo, Japan). Low-density lipoprotein (LDL) cholesterol levels were determined by a direct quantitative assay, and serum phosphate levels were determined by an enzymatic assay. These biochemical data were analyzed using an automated analyzer (AU800, Olympus Corp., Tokyo, Japan). Plasma glucose levels were determined by an enzymatic assay using an automated analyzer (H-7150 Hitachi High-Technology Corp., Tokyo, Japan). Glycosylated hemoglobin (HbA_1c_) levels were determined by a latex agglutination turbidimetric immunoassay using an automated analyzer (JCA-BM9030, JEOL Ltd, Tokyo, Japan). Serum levels of C-reactive protein (CRP) were determined by the latex-enhanced immunonephelometric method (Dade Behring Diagnostic, Germany). Combined blood cell counts were determined using automated blood cell counters (Sysmex XE-2100 and Sysmex SE-9000, Sysmex, Kobe, Japan). Determinations of total cholesterol levels and HDL cholesterol levels were performed under the quality control program of the Centers for Disease Control and Prevention in the United States through the Osaka Medical Center for Health Science and Promotion, Japan.^6^

### Data Handling and Classification

We determined causes of ESRD and comorbid conditions primarily according to diagnostic criteria (Table 1).^7^^,^^8^ To compare characteristics of patients with similar causes of ESRD, patients were divided into three groups: a chronic glomerulonephritis group, a diabetic nephropathy group, and an other renal diseases group.

**Table 1.** Criteria for determining the causes of end-stage renal disease and comorbid conditions in hemodialysis patients.

Causes of end-stage renal disease		Comorbid conditions	
Chronic glomerulonephritis (CGN)	1 Hematuria 2 Proteinuria (2+, 3+) 3 Sustained renal insufficiency The diagnosis of CGN required that all three above-mentioned criteria or pathology be diagnosed by biopsy.	Myocardial infarction	1 Evolving Q-wave (at least 2 lead) myocardial infarction 2 Cardiac enzymes elevation: more than twice the normal range 3 Sustained chest pain lasting at least 30 minutes The diagnosis of myocardial infarction required two of the above-mentioned criteria.
Diabetic nephropathy (DMN)	1 Clinically diagnosed as diabetes mellitus 2 Proteinuria (≥ 300 mg/day) or edema or hypertension or renal insufficiency The diagnosis of DMN required that both above-mentioned criteria or pathological diagnosis be confirmed by biopsy.	Peripheral arterial disease	1 History of bypass surgery or angioplasty 2 Ankle-arm systolic blood pressure ratio of ≤ 0.8. 3 Exertional leg pain relieved by rest plus claudication diagnosed by physician. The diagnosis of peripheral artery disease required one of the above-mentioned criteria or image modality identification.
Hypertensive nephrosclerosis	1 Proteinuria (±, +) 2 Hypertension 3 Sustained renal insufficiency The diagnosis of hypertensive nephrosclerosis required that all above-mentioned 3 criteria or pathological diagnosis by biopsy.	Stroke	1 Abrupt onset of new neurologic deficit lasting at least 24 hours, with specific localizing findings comfirmed by physician 2 Without evidence for underlying nonvascular cause. The diagnosis of stroke required both 1 and 2 criteria or image modality identification (CT or MRI).
Polycystic kidney disease	The diagnosis of polycystic kidney disease required that image modalities (CT, US or MRI) identity multiple cysts in both kidneys.	Hypertension (HTN)	1 Anti-hypertension medication 2 Systolic blood pressure ≥ 140 mmHg 3 Diastolic blood pressure ≤ 90mm Hg The diagnosis of HTN required one of the above-mentioned criteria
Lupus nephritis	1 Clinically diagnosed as systemic lupus erythematosus Sustained renal insufficiency 2 The diagnosis of lupus nephritis required that both above-mentioned criteria and pathological diagnosis be confirmed by biopsy.	Diabetes mellitus (DM)	1 Past or current use of hypoglycemic agents 2 Casual plasma glucose ≥ 200 mg/dL 3 HbA_1c_ ≥ 6.5% The diagnosis of DM required one of the above-mentioned criteria.
		Dyslipidemia	1 Past or current use of anti-hyperlipidemia agents 2 Serum total cholesterol level ≥ 220 mg/dL 3 Serum low-density lipoprotein cholesterol level ≥ 140 mg/dL 4 Serum high-density lipoprotein level ≤ 40 mg/dL The diagnosis of dyslipidemia required at least one of the above-mentioned criteria.

CT: computed tomography

US: ultrasonography

MRI: magnetic resonance imaging

Habitual smoking was defined as currently smoking. Regular alcohol drinking was defined as drinking five or more days per week. Pulse pressure (PP) was defined as the difference between SBP and DBP, and delta SBP was defined as pre-dialysis SBP minus post-dialysis SBP. We defined persons with HDL cholesterol levels of less than 40 mg/dL as persons with low HDL cholesterol levels. We defined persons with CRP levels of more than 10 mg/L as persons with high CRP levels.

The Iwate KENCO Study is a population-based study that has been carried out in the general population in the same area as the KAREN Study.^9^^,^^10^ We compared CVD risk factors and cardiovascular comorbid conditions in hemodialysis patients in the KAREN Study to those in the general population, as determined in the Iwate KENCO Study.

### Statistical Analysis

Continuous variables are expressed as means ± standard deviation, and the Student’s t test or the chi square test was used to compare two groups. The Mann-Whitney U test was used for skewed data (TG levels and CRP levels). One-way analysis of variance (ANOVA) or the Kruskal-Wallis test (TG levels and CRP levels) was used to compare three or more groups. Multiple comparisons were performed using Bonferroni’s method, and age-adjusted values were calculated by the direct method based on data from the Iwate KENCO Study. Standardized morbidity ratios (SMRs) of myocardial infarction, stroke, hypertension, and diabetes mellitus in hemodialysis patients were also calculated based on data from the Iwate KENCO Study.

A p-value of less than 0.05 was considered statistically significant. All statistical analyses were performed using SPSS(r) software (SPSS, Japan Inc., Version 11.0).

## RESULTS

Table 2 shows patient characteristics. The mean age of the 1,214 patients (779 males and 435 females) was 61.2 ± 13.0 years (range, 22 to 95 years). The mean age at the start of hemodialysis was 54.2 ± 15.8 years, and the mean duration of hemodialysis was 7.0 ± 6.7 years. These numbers are similar for both male and female patients. Mean BMIs were 21.2 ± 2.9 in the male patients and 20.2±3.1 in the female patients.

**Table 2.** Characteristics of patients in the KAREN Study.

		Total	Male	Female	p-value
Number		1214	779	435	
Age	(year)	61.2 ± 13.0	61.1 ± 13.1	61.4 ± 12.7	NS
Age at starting hemodialysis	(year)	54.2 ± 15.8	54.1 ± 16.0	54.3 ± 15.3	NS
Body Mass Index	(kg/m^2^)	20.8 ± 3.0	21.2 ± 2.9	20.2 ± 3.1	< 0.001
Duration of hemodialysis	(year)	7.0 ± 6.7	6.9 ± 6.9	7.1 ± 6.5	NS
Sessions of hemodialysis	(/week)	2.88 ± 0.35	2.89 ± 0.33	2.85 ± 0.39	NS
Length of a hemodialysis session	(hour)	3.73 ± 0.64	3.80 ± 0.65	3.62 ± 0.54	< 0.001
Cause of end-stage renal disease	(%)				
Glomerulonephritis		29.8	29.1	31.0	NS
Diabetic nephropathy		24.5	27.5	19.3	0.002
Hypertensive nephrosclerosis		9.8	9.9	9.7	NS
Polycystic kidney		3.5	3.2	4.1	NS
Other minor diseases		7.4	6.4	9.2	0.036
Unknown		24.9	23.9	26.7	NS
Comorbid condition					
Myocardial infarction		5.2	5.4	4.8	NS
Stroke		13.1	13.1	13.1	NS
Peripheral artery disease		16.1	16.2	16.1	NS
Habits	(%)				
Currently smoking		28.2	39.5	7.8	< 0.001
Regular drinking		6.9	9.1	3.0	< 0.001
Hypertension	(%)	87.1	88.2	85.3	NS
Anti-hypertension medication	(%)	68.5	70.6	64.6	0.036
Number of prescribed drugs		1.36	1.41	1.28	0.070
Pre-systolic blood pressure	(mmHg)	155 ± 24	155 ± 23	155 ± 25	NS
Pre-diastolic blood pressure	(mmHg)	85 ± 13	85 ± 14	85 ± 134	NS
Post-systolic blood pressure	(mmHg)	142 ± 26	143 ± 25	140 ± 28	0.041
Post-diastolic blood pressure	(mmHg)	80 ± 14	80 ± 14	79 ± 14	NS
Delta-systolic blood pressure	(mmHg)	13 ± 23	11 ± 22	15 ± 23	0.012
Diabetes mellitus	(%)	29.1	32.1	23.7	< 0.001
HbA_1c_	(%)	4.68 ± 0.95	4.69 ± 0.93	4.65 ± 0.98	NS
Plasma glucose	(mg/dL)	128.3 ± 54.8	129.6 ± 57.6	126.0 ± 49.4	NS
Dyslipidemia	(%)	43.1	46.1	37.2	< 0.001
Total cholesterol	(mg/dL)	154.9 ± 35.6	148.1 ± 33.6	166.9 ± 36.0	< 0.001
Triglyceride	(mg/dL)	108.6 ± 67.7	106.6 ± 72.3	112.3 ± 58.3	< 0.001*
High-density lipoprotein (HDL) cholesterol	(mg/dL)	47.0 ± 15.3	45.1 ± 14.9	50.4 ± 15.4	< 0.001
Low-density lipoprotein (LDL) cholesterol	(mg/dL)	84.9 ± 27.0	81.0 ± 26.2	91.8 ± 26.9	< 0.001
% of low HDL cholesterol (< 40mg/dL)	(%)	35.9	42.0	25.1	< 0.001
Nutrition-related data					
Total protein	(g/dL)	6.5 ± 0.5	6.5 ± 0.5	6.4 ± 0.5	0.001
Serum albumin	(g/dL)	3.7 ± 0.4	3.8 ± 0.4	3.7 ± 0.4	0.011
Blood urea nitrogen	(mg/dL)	71.2 ± 15.7	70.9 ± 15.4	71.8 ± 16.1	NS
Serum creatinine	(mg/dL)	11.0 ± 2.8	11.5 ± 3.0	10.1 ± 2.2	< 0.001
Inflammatory markers					
White blood cell count	(/µL)	5732 ± 1739	5891 ± 1765	5446 ± 1654	< 0.001
C-reactive protein (CRP)	(mg/L)	4.01 ± 9.26	4.27 ± 8.40	3.54 ± 10.62	NS*
% of high CRP (> 10 mg/L)		9.4%	10.7%	7.0%	0.025

Data are expressed as means ± standard deviation, or percentages.

P-values were obtained by a Student’s t test, the chi square test, or the Mann-Whitney U test (triglyceride levels and CRP levels).

*: p-values by the Mann-Whitney U test.

The most common cause of ESRD was chronic glomerulonephritis (29.8%), and the second-most common cause was diabetic nephropathy (24.5%). The etiology was unknown in 24.9% of the patients. The proportions of patients with myocardial infarction, stroke, and peripheral arterial disease were 5%, 13%, and 16%, respectively. The proportions of smokers were 39.5% of male patients and 7.8% of female patients, while 9.1% of the male patients and 3.0% of the female patients were regular alcohol drinkers.

The majority (87.1%) of patients had hypertension, and 78.6% of the patients with hypertension took anti-hypertension medication. Pre-dialysis SBP and DBP were similar in the male patients and female patients. Post-dialysis SBP in the female patients was significantly lower than that of the male patients. About one-third (29.1%) of the patients had diabetes mellitus; the percentage of male patients with diabetes mellitus was higher than that of female patients with diabetes mellitus, but the mean levels of plasma glucose and HbA1c were similar in both male and female patients. The proportion of patients with dyslipidemia was 43.1%, and 83.1% of the patients with dyslipidemia had low HDL cholesterol levels. Mean levels of total cholesterol, LDL cholesterol, HDL cholesterol, and triglycerides in the female patients were higher than the corresponding levels in male patients. Mean levels of CRP were 4.27 mg/L in the male patients and 3.54 mg/L in the female patients. CRP levels were higher than 10 mg/L in 10.7% of the male patients and in 7.0% of the female patients.

Table 3 shows characteristics of patients in the three renal disease groups. The mean age at the beginning of hemodialysis in the diabetic nephropathy group was higher than the mean ages of the other two groups, and the mean BMI and percentage of male patients in the diabetic nephropathy group were higher than those of the other two groups. Mean duration of hemodialysis differed between the three groups, with the shortest in the diabetic nephropathy group and the longest in the chronic glomerulonephritis group.

**Table 3.** Characteristics of patients in groups according to cause of end-stage renal disease.

	Chronic glomerulonephritis (a)		Diabetic nephropathy (b)	Others ©	p-value	multiple comparisons or χ^2^ test			

						a vs b		a vs c	b vs c
Number		362	298	554					
Male/Female		227 / 135	214 / 84	338 / 216	0.006	*			*
Age	(year)	57.7 ± 12.9	62.8 ± 11.0	62.5 ± 13.6	< 0.001	**		**	
Age at starting hemodialysis	(year)	48.1 ± 15.9	59.2 ± 11.3	55.5 ± 16.6	< 0.001	**		**	**
Body Mass Index	(kg/m^2^)	20.5 ± 2.8	21.3 ± 3.0	20.8 ± 3.1	0.002	**			**
Duration of hemodialysis	(year)	9.6 ± 7.7	3.7 ± 3.3	7.1 ± 6.7	< 0.001	**		**	**
Sessions of hemodialysis	(/week)	2.91 ± 0.33	2.84 ± 0.37	2.87 ± 0.36	NS				
Length of a hemodialysis session	(hour)	3.80 ± 0.61	3.69 ± 0.63	3.71 ± 0.62	0.039	*			

Comorbid condition	(%)								
Myocardial infarction		5.5	4.4	5.4	NS				
Stroke		10.8	14.1	14.1	NS				
Peripheral artery disease		19.1	15.1	14.8	NS				

Hypertension	(%)	83.4	95.3	85.2	< 0.001	*			*
Anti-hypertension medications	(%)	63.8	82.9	63.7	< 0.001	*			*
Number of prescribed drugs		1.23 ± 1.18	1.78 ± 1.29	1.23 ± 1.18	< 0.001	**			**
Pre-systolic blood pressure	(mmHg)	150 ± 23	166 ± 25	152 ± 22	< 0.001	**			**
Pre-diastolic blood pressure	(mmHg)	86 ± 13	85 ± 13	84 ± 14	0.048			**	
Pre-pulse pressure	(mmHg)	64 ± 16	81 ± 18	68 ± 16	< 0.001	**			**
Post-systolic blood pressure	(mmHg)	137 ± 26	153 ± 27	140 ± 24	< 0.001	**			**
Post-diastolic blood pressure	(mmHg)	80 ± 15	80 ± 13	79 ± 14	NS				
Post-pulse pressure	(mmHg)	56 ± 16	73 ± 19	60 ± 13	< 0.001	**			**
Delta-systolic blood pressure	(mmHg)	13 ± 20	13 ± 25	12 ± 22	NS				

Diabetes mellitus	(%)	5.2	100	6.5	< 0.001	*			*
HbA_1c_	(%)	4.34 ± 0.61	5.60 ± 1.13	4.41 ± 0.66	< 0.001	**			**
Plasma glucose	(mg/dL)	111.8 ± 35.5	169.6 ± 69.8	116.9 ± 43.8	< 0.001	**			**

Dyslipidemia	(%)	43.1	56.4	41.3	< 0.001	*			*
Total cholesterol	(mg/dL)	155.1 ± 32.1	152.9 ± 37.9	155.8 ± 36.5	NS				
Triglyceride	(mg/dL)	109.3 ± 59.2	116.7 ± 81.2	103.8 ± 64.4	0.024^†^	‡			‡
High-density lipoprotein (HDL) cholesterol	(mg/dL)	47.4 ± 16.2	44.5 ± 14.4	48.0 ± 15.0	0.005	**			**
Low-density lipoprotein (LDL) cholesterol	(mg/dL)	84.7 ± 25.0	83.7 ± 27.5	85.6 ± 27.9	NS				
% of low HDL cholesterol (< 40mg/dL)	(%)	36.7	44.0	31.0	< 0.001				*
Habits									
Currently smoking	(%)	28.4	29.2	27.5	NS				
Regular drinking	(%)	9.1	7.0	5.4	NS				
Nutrition-related data									
Total protein	(g/dL)	6.5 ± 0.5	6.5 ± 0.5	6.5 ± 0.5	NS				
Serum albumin	(g/dL)	3.8 ± 0.4	3.7 ± 0.4	3.8 ± 0.4	0.001	**			**
Blood urea nitrogen	(mg/dL)	72.1 ± 14.8	68.7 ± 15.4	71.9 ± 16.2	0.007	**			**
Serum creatinine	(mg/dL)	11.8 ± 2.8	9.8 ± 2.5	11.2 ± 2.7	< 0.001	**			**
Inflammatory markers									
White blood cell count	(/µL)	5682 ± 1783	6087 ± 1658	5572 ± 1728	< 0.001	**			**
C-reactive protein (CRP)	(mg/L)	3.87 ± 9.56	4.43 ± 9.36	3.87 ± 900	NS^†^				
% of high CRP (> 10 mg/L)	(%)	9.1	11.1	8.5	NS				

Data are expressed as means ± standard deviations, or as percentages.

P-values were obtained by ANOVA, the chi square test, or the Kruskal-Wallis test (†).

*: p< 0.05, **: p < 0.01, by the multiple comparison test (Bonferroni method) or the chi square test.

In the diabetic nephropathy group, all blood pressure parameters except for DBP remained high regardless of intensive anti-hypertension medication. The percentage of patients with dyslipidemia and the mean levels of triglycerides were higher, and the mean levels of HDL cholesterol were lower, in the diabetic nephropathy group relative to the other two groups. Nutrition-related parameters such as mean levels of serum albumin, BUN, and creatinine were lower in the diabetic nephropathy group than in the other groups. The mean white blood cell count in the diabetic nephropathy group was higher than in the other two groups.

Table 4 compares comorbid conditions, BMI, blood pressure, lipid levels, albumin levels, and CRP levels in hemodialysis patients to those of the general population. SMRs of myocardial infarction and stroke were 9.6 (8.0 in males and 28.2 in females) and 5.7 (3.6 in males and 8.3 in females), respectively. Mean levels of total serum cholesterol, HDL cholesterol, albumin, and BMI in the ESRD patients were lower than in the general population. Both the mean levels of CRP and the percentage of patients with high CRP levels were higher than in the general population.

**Table 4.** Comparison of prevalence, comorbidity, anthropometrical data, and blood sampling data in end-stage renal disease (ESRD) patients in the KAREN Study with those of the general population, using the Iwate KENCO Study.

		Male				Female				Total			
		
		General population	ESRD patients			General population	ESRD patients			General population	ESRD patients		
Number		4029		779		7338		435		11367		1214	
Comorbidity													
		prevalence	prevalence	SMR	(95% CI)	prevalence	prevalence	SMR	(95% CI)	prevalence	prevalence	SMR	(95% CI)
Myocardial infarction		0.81%	5.5%	8.0	(5.6, 10.4)	0.18%	4.9%	28.2	(16.2, 40.3)	0.40%	5.2%	9.6	(6.7, 12.3)
Stroke		4.05%	13.1%	3.6	(2.9, 4.4)	1.66%	13.1%	8.3	(6.1, 10.5)	2.50%	13.1%	5.7	(4.8, 6.6)
Hypertension		23.0%	88.2%	4.3	(4.0, 4.6)	23.2%	85.3%	3.8	(3.4, 4.2)	23.2%	87.1%	4.0	(3.8, 4.3)
Diabetes mellitus		6.92%	32.1%	5.1	(4.5, 5.7)	3.65%	23.7%	6.8	(5.5, 8.1)	4.81%	29.5%	6.5	(5.8, 7.2)
% high C-reeactive protein (> 10mg/L)		1.44%	10.9%	8.4	(6.6, 10.2)	1.17%	6.99%	6.1	(3.9, 8.3)	1.27%	9.47%	7.8	(6.4, 9.3)

Anthopometrical and blood sampling data (mean value)													
		Age-adjusted mean				Age-adjusted mean				Age- and sex-adjusted mean			
Body Mass Index	(kg/m^2^)	23.7	21.2			24.0	20.1			23.9	20.5		
Systolic blood pressure	(mmHg)	130	155			126	155			127	155		
Diastolic blood pressure	(mmHg)	77	85			74	85			75	85		
Total cholesterol	(mg/dL)	193.5	149.1			208.1	166.9			203.3	160.6		
HDL cholesterol	(mg/dL)	56.2	45.1			61.5	50.4			59.7	48.5		
Serum albumin	(g/dL)	4.4	3.8			4.4	3.7			4.4	3.7		
C-reactive protein	(mg/L)	1.39	4.27			1.04	3.54			1.16	3.80		

Data are expressed as means or percentages or standardized morbidity ratios (SMRs).

SMR: standardized morbidity ratios

CI: confidence interval

## DISCUSSION

The KAREN Study was designed as a population-based prospective study to assess the effects of risk factors on CVD morbidity and mortality in ESRD patients under a quality control program, and the study covered more than 80% of hemodialysis patients in the area of interest. Analysis of baseline data from the KAREN Study revealed CVD risk factors and cardiovascular comorbidities in hemodialysis patients.

About 90% of the hemodialysis patients in the KAREN Study had hypertension, and 78.6% of the hypertensive patients took anti-hypertension medications. More than 40% of the patients in the KAREN Study had dyslipidemia. More than 80% of patients with dyslipidemia had low HDL cholesterol levels, a similar percentage to that found in a previous study.^11^

Diabetic nephropathy accounted for 25% of all causes of ESRD and 29% of the KAREN Study patients had diabetes mellitus. The proportion of patients with hypertension and mean WBC count were higher, and levels of nutrition-related markers were lower, in the diabetic nephropathy group than in the other two groups. These conditions may be related to the poor prognoses for patients with diabetic nephropathy.^12^^,^^13^

The Okinawa Dialysis Study (OKIDS) revealed cardiovascular comorbid conditions in ESRD patients more than ten years ago,^4^^,^^5^ but the prevalence of cardiovascular comorbidity for each renal disease subgroup was not shown. One benefit of our study was that it revealed comorbid conditions of hemodialysis patients for each renal disease group. The prevalences of myocardial infarction and stroke were similar between the renal disease groups. The cardiovascular comorbidities in the diabetic nephropathy patients were not different from those in patients with other renal diseases, a finding that disagrees with the results of an ESRD study in the United States.^13^ The percentage of smokers in the KAREN Study (28%) is reflective of the general population of Japan.^9^^,^^14^ Further studies are needed to determine whether smoking contributes to the high risk of CVD in ESRD patients in Japan, and efforts should be increased to encourage ESRD patients to stop smoking.^15^

The percentage of patients with diabetic nephropathy in the KAREN Study was similar to that of the Japanese Society for Dialysis Therapy (JSDT) Survey.^1^ However, the proportion of patients with chronic glomerulonephritis in the KAREN Study was lower, and the percentage of patients with hypertensive nephrosclerosis in the KAREN Study was higher, than those in the JSDT Survey.^1^

In the current study, we identified causes of ESRD using information from medical records. The most common reason for classifying patients as unknown etiology was insufficient information regarding whether onset of proteinuria preceded that of hypertension. The low rate of diagnostic renal biopsy (10.4%) also made differential diagnosis difficult. Thus, it is possible that some patients with chronic glomerulonephritis or hypertensive nephrosclerosis should have been classified as patients with unknown etiology.

The percentage of patients with chronic glomerulonephritis was higher, and the percentage of patients with hypertensive nephrosclerosis was lower in the KAREN Study than those reported by United States Renal Data System (USRDS).^2^ The prevalence of hypertension was similar, the prevalence of myocardial infarction was lower, and the prevalence of stroke was higher in the KAREN Study. These results seem to reflect a high prevalence of stroke and a low prevalence of myocardial infarction in the Japanese general population relative to the general American population.^16^^,^^17^

In this study, the prevalence of CVD comorbidities was higher, albumin levels were lower, and CRP levels were higher in hemodialysis patients than in the general population. The lower albumin levels in hemodialysis patients may contribute to the high incidence of CVD.^18^ Serum CRP levels in hemodialysis patients were significantly higher than those in the general population (1.80 mg/L vs. 0.84 mg/L) even after removal of subjects with apparently elevated CRP levels (10+ mg/L), and the high risk for CVD in hemodialysis patients might be partly explained by the large percentage of subjects with low-grade inflammation.^19^^-^^22^

It has been shown that traditional risk factors were not associated with the development of CVD in hemodialysis patients.^23^ Some authors reported that malnutrition, inflammation, and atherosclerosis were closely linked in ESRD patients, and suggested that malnutrition and inflammation are stronger predictors than are traditional risk factors for hemodialysis patients.^23^^,^^24^

Instead of collecting isolated cases, we collected prevalent cases of hemodialysis in our study. This approach may fail to detect cases that are more serious. We were unable to make appointments with 52 patients because of serious physical conditions, and initial investigations were not conducted for 21 patients because of deteriorated health. Patients who would not give informed consent were probably in poorer condition. These factors might have reduced the number of serious cases of ESRD in our study; thus, the results obtained of our study might represent results for ESRD patients in relatively good condition. The prevalence of risk factors and comorbidities might therefore be underestimated.

We compared comorbid conditions in ESRD patients with the general population. In the Iwate KENCO Study, comorbid conditions were assessed using self-reported questionnaires, while in the KAREN Study, they were assessed using patients’ medical records. This difference may artificially exaggerate differences in the prevalences of comorbidities between hemodialysis patients and the general population.

In conclusion, hemodialysis patients have a high prevalence of cardiovascular risk factors and comorbidities. Levels of nutrition-related markers were lower, and CRP levels were higher, in hemodialysis patients relative to the general population.

## APPENDIX

The KAREN Study Group

Yahaba Clinic: Mikihiko Fujishima, Yuko Nakamura, Toshiko Kumagai; San-ai Hospital: Kohei Yamauchi, Koji Seino, Tayo Kambayashi; Yuai Hospital: Shigeru Nagasawa, Akira Suzuki; Yamada Clinic: Ikuo Yamada; Isurugi Clinic: Takashi Isurugi; Morioka Red Cross Hospital: Teruo Kagabu, Susumu Terasato; Ohinata Clinic: Mitsuru Ohinata; Numakunai Clinic: Jun-ichi Matsuzaka; Ninohe Clinic: Hikaru Aoki; Iwate Prefectural Ichinohe Hospital: Masato Ozawa, Tadao Toda; Iwate Prefectural Kuji Hospital: Ikuhiko Yoshida, Takuji Kaneko; Taneichi Hospital: Kiyoshi Urushikubo, Tamako Kasatsuki; Obara Clinic: Noriaki Obara, Nobuko Miyakawa, Masako Takahashi; Iwate Rosai Hospital: Kazuo Murai, Tsuneo Kajikawa; Hoyo Hospital: Takao Ishihara, Satsu Utsunomiya, Toshihiro Sato; Iwate Prefectural Kitakami Hospital: Katsuya Goto, Kazuo Noro; Kitakami Saiseikai Hospital: Masataka Abe, Kaoru Suzuki; Hidakami Chuo Clinic: Shigetoshi Kanazawa; Kitakami Jin Clinic: Hiroyuki Koike; Sawauchi Hospital: Toshimichi Sato, Fusanori Kunugita; Iwate Prefectural Miyako Hospital: Ken-ichi Nagai, Hiroshi Takagane; Goto Urologic Clinic: Yasufumi Goto, Yasuki Goto, Takanao Tanaka; Goto Clinic: Takashi Goto; Iwaizumi Saiseikai Hospital: Yoshihiro Shibano, Yoshiyuki Yashima.
